# Weight Loss Challenges in Achieving Transplant Eligibility in Patients With Kidney Failure: A Qualitative Study

**DOI:** 10.1016/j.xkme.2021.09.005

**Published:** 2021-11-11

**Authors:** Johanne Freeman, Hanne Konradsen, Kristine Lindhard, Ditte Hansen

**Affiliations:** 1 Department of Gastroenterology, Herlev and Gentofte Hospital, Herlev, Denmark; 2 Department of Nephrology, Herlev and Gentofte Hospital, Herlev, Denmark; 3 Department of Clinical Medicine, Faculty of Health and Medical Sciences, Herlev, Denmark; 4 University of Copenhagen, Herlev, Denmark; 5 Karolinska Institutet, NVS, Solna, Sweden

**Keywords:** Chronic kidney disease, obesity, overweight, quality of life, qualitative research

## Abstract

**Rationale & Objective:**

Patients with kidney failure need kidney replacement therapy to maximize survival. Kidney transplant is a superior mode of kidney replacement therapy for most individuals with kidney failure. Patients with obesity often are not approved for kidney transplant until they lose sufficient weight, as obesity may complicate the surgical procedure, and the risk of graft loss increases with a higher body mass index. To help potential kidney transplant recipient candidates lose weight, further knowledge of their thoughts, feelings, and attitudes is needed.

**Study Design:**

Qualitative study with semistructured interviews and an exploratory research design, guided by qualitative content analysis.

**Setting & Participants:**

Patients at a hospital in Denmark required to lose weight to achieve kidney transplant eligibility.

**Analytical Approach:**

From patients’ responses, we identified descriptive themes using a phenomenological approach. The factors affecting outcomes were derived reflexively from these themes.

**Results:**

Ten interviews were analyzed. Experiences of obesity and weight-loss attempts were described across 4 themes; (1) restrictions and exhaustion, (2) hope and hopelessness, (3) support and self-discipline, and (4) motivation based on severity. A major motivating factor to achieving weight loss in the studied group of patients was their declining kidney function and the fact that kidney transplant cannot be considered until sufficient weight loss is achieved.

**Limitations:**

Thematic saturation was reached after an unexpectedly low number of participants. The patients were only interviewed once and over the phone.

**Conclusions:**

Patients with obesity who are seeking kidney transplant need additional help with the dietary restrictions brought on by kidney disease. They need assistance bridging between a kidney-friendly diet and a sustainable diet that will ensure weight loss. These patients also express not wanting to feel alone in their weight-loss battle. They are looking for help and support to achieve weight loss.

**Plain-Language Summary**

Patients with kidney failure who are overweight must lose weight to qualify for a kidney transplant. Kidney disease is known to have widespread dietary restrictions that are often contradictive to a diet needed to lose weight. Additionally, kidney disease is known to cause fatigue, making exercise to promote weight loss challenging. In this qualitative study, we interviewed participants to explore their thoughts on these issues and find out what specifically they think makes it so difficult to lose weight and what clinicians can do to make it easier for them.

## Introduction

In Denmark, it is estimated that 10%-15% of the adult population has kidney disease.^1^ Chronic kidney disease (CKD) can arise from a number of diseases, most commonly diabetes and hypertension.^1^ Progressive loss of kidney function occurs and ultimately results in the need for kidney replacement therapy (dialysis or transplant).^2^ For suitable candidates, kidney transplant is preferred over dialysis because it increases the rate of survival, improves the quality of life, and is less costly than dialysis.^3^^,^^4^ In patients with obesity, pretransplant weight loss is necessary to reduce the risk of surgical complications at the time of transplant and improve the outcome of graft function after transplant.^5^, ^6^, ^7^, ^8^ Patients with a body mass index (BMI) ranging from 30-34.9 kg/m^2^ have an improved graft survival rate compared with patients with a BMI ≥35 kg/m^2^.^9^ Obesity has become both a driver for the increased incidence of kidney failure and a barrier to treatment of patients with kidney failure.^10^ The prevalence of obesity worldwide has shown a marked increase from the 1980s to the 2000s.^11^ In the United States, between 2012 and 2014, nearly 40,000 dialysis patients had obesity as the sole contraindication to wait-listing for kidney transplant. A higher BMI has been associated with a lower likelihood of listing, and of those with BMI 35-39.9 kg/m^2^ who were already listed, 24% were less likely to actually achieve transplant.^12^ Previous studies have recommended bariatric surgery to obtain sufficient weight loss in patients with kidney failure,^5^^,^^13^^,^^14^ and other studies have suggested that patients treated with dialysis with obesity need intensive weight-loss counseling to prepare for a kidney transplant.^15^ Studies have shown that these patients are well aware of their obesity and the need to lose weight but feel unable to do so.^16^ Thus, attention should be paid to how clinicians can support long-term weight loss in these patients. This study aims to explore the thoughts, feelings, and attitudes of patients with obesity who must lose weight to receive a new kidney. The hope is that we as health professionals can adapt and find better ways to help patients with obesity achieve adequate and sustainable weight loss.

## Methods

### Design

This semistructured interview study used an exploratory research design, guided by qualitative content analysis.^17^, ^18^, ^19^

### Data Collection

Data were collected in the fall of 2020. For the sampling of participants, maximal variation was desired, including age, different causes of CKD, female and male sex, patients receiving dialysis, or patients with estimated glomerular filtration rate <15 mL/min/1.73 m^2^ who were not yet receiving dialysis. The patients needed to be able to communicate in Danish. At the time of the interviews, the patients were associated with departments of nephrology at 2 medium-sized hospitals. As the data collection took place during the COVID-19 pandemic, patients who agreed to participate were interviewed over the telephone at a time convenient for the patient. The researcher repeated the information about the study and obtained consent from the patients verbally and in writing. A semistructured interview guide was used (Box 1). Questions were developed based on existing literature and the aim of the study. The questions were open-ended and revolved around the patients’ experience of being overweight, the impact this has had on their everyday life, the level of support they felt in their weight-loss attempts, and their long-term goals for weight loss. The interviews ranged from 7 to 20 minutes, with the average being 13 minutes. The interviews were tape-recorded and transcribed verbatim. Data were saved to a OneDrive folder provided by The University of Copenhagen.

Box 1 **Interview Questions Based on Existing Literature**

• Tell me about the entire course of your disease.
• If you go all the way back to your childhood and until today, how has your relationship with your body and weight been?
• How has your relationship with food been throughout your life? Has your weight gone up and down?
• How has your relationship with physical activity been throughout your life? Have you been physically active throughout your life?
• Is it your experience that your family and friends understand your situation and can offer you the support you need?
• How do you plan to succeed in losing weight?
• How has your experience been having a kidney illness and having been met with restrictions concerning your weight, to receive treatment?
• How much weight do the doctors want you to lose?
• What kind of support do you think would be helpful in losing weight? Have you received any support, and was it helpful?
• Do you believe that you will be successful in losing weight?

### Data Analysis

Interviews were transcribed shortly after having taken place. The transcriptions were then analyzed using inductive conventional content analysis.^17^ The purpose was to derive meaning from the interviews and identify recurring conceptual patterns of experience across the data material. The analysis took place in phases. First, the author listened through the audio recordings and carefully read and re-read the transcriptions to become familiar with the content of each interview. The next step was identifying meaning units by collecting words or statements relating to the same central meaning. The meaning units were condensed and sorted into themes related to their content. Data collection ended when saturation was reached, which was determined to be when no new information was being gained from the interviews. Throughout the analytical process, the meaning units and themes were discussed by the authors to increase the validity and trustworthiness of the results.^19^

### Ethical Approval

The patients were informed that participation was entirely voluntary and that they could withdraw at any point for any reason. They were guaranteed confidentiality and anonymity in the presentation of the findings. Ethical approval was not necessary according to Danish law because this was an interview study.^20^ This study was part of a Master’s thesis, which is protected under the data laws of The University of Copenhagen.

## Results

### Participants

A total of 10 patients from the same outpatient clinic at Herlev Hospital were invited to participate, and all of them were included in the study; 7 men and 3 women, with an age range of 42-66 years (Table 1). Seven of the patients were already receiving dialysis and 3 were not. Nine of these patients were attempting to lose weight to meet the criteria for being approved for the kidney transplant list and one patient had lost sufficient weight and had already been added to the list.

**Table 1:** Summary Characteristics of the Study Population

Demographic Data	
Total number of participants	10
Sex	
Female	3 (30)
Male	7 (70)
Age, y	54 (42-66)
Body mass index, kg/m^2^	39.4 (29.3-51.2)
Dialysis	7 (64)
Diabetes	4 (40)
Marital status	
Single	2 (20)
Married/de facto	8 (80)
Employment status	
Employed	5 (50)
Retired/early retirement	5 (50)

*Note:* Data are presented as numbers (percentages) or median (range).

Four themes came to light during the analytic process of the interviews. Table 2 provides an example of the analytical process with examples of quotes in each theme. The theme restrictions and exhaustion describes how the patients handle strict dietary restrictions and the struggle with exhaustion brought on by obesity and kidney disease. The theme hope and hopelessness captures how patients who are trying to lose weight feel a deep sense of hopelessness. Most of the time they are on the brink of giving up, yet, in the middle of the hopelessness, they discover new hope and renewed energy to push on. The theme support and self-discipline expresses how patients want support from the hospital, general practitioner, and local community, along with family and friends, but how they also discover that they are the only ones responsible to make a change. The last theme, motivation based on severity, describes how patients tend to put off weight loss until their condition is so dire that they are forced to act on it.

**Table 2:** Examples of the Analytic Process

Illustrative Quote	Meaning Unit	Category
“I feel like many of the things I need to eat in relation to my kidney disease, are the same things, they say, don’t work on a diet, such as white bread, which you would normally avoid if you’re on a diet.” “I need to find a way to deal with the exhaustion, that’s something I need to find a rhythm in before I can add exercise to my schedule.”	Confusion about what to eat in addition to general exhaustion makes weight loss hard.	Restrictions and exhaustion
“When you have to lose more than 20 kg you might as well give up right away. It’s not realistic, and not something you just do in 6 months… I find it entirely impossible.” “I have actually already lost 12 kg, so I’m counting on that I can get on the list pretty soon.”	The mindset of the participant was instrumental in achieving or not achieving weight-loss.	Hope and hopelessness
“I was extremely frustrated when there was no help to get, but then I said, OK, only you can do something about this.”	Support is expected in trying to achieve weight-loss, but it is also understood that it’s the responsibility of the individual.	Support and self-discipline
“It wasn’t until I started in dialysis that it became serious to me, when you suddenly can’t do anything at all, it’s time to get in gear.”	Illustrates how the seriousness of a disease influences motivation.	Motivation based on severity

### Restrictions and Exhaustion

Patients described struggling with the dietary restrictions kidney disease brings while also having to lose weight. Several patients expressed frustrations about the restricted diet. One participant said, “It’s extremely hard to lose weight when you have to be careful all the time and think about how much potassium and phosphate is in the different foods” (P2). Another interviewee said, “I have tried to eat reasonably, but I find it difficult since there are so many things I can’t eat” (P4). A patient who has diabetes in addition to kidney disease said, “One thing is to have this diabetes, and I used to be allowed to eat fresh vegetables. But then when I got my kidney disease, I found it very difficult that I couldn’t eat that anymore” (P9). Another participant said, “I feel like many of the things I need to eat in relation to my kidney disease, are the same things, they say, don’t work on a diet, such as white bread, which you would normally avoid if you’re on a diet” (P4).

The participants in this study described an extreme level of exhaustion. Many expressed that the energy simply was not there to get out and exercise. One participant said, “I was so tired after getting off work, that I had to go home and sleep for an hour before I could go and do sports” (P6). Another said he wished to have more energy. One participant believed that his obesity was the main reason for his tiredness: “You get extremely hindered from particularly extreme obesity, you’re constantly tired, and adding a kidney disease and dialysis on top of that doesn’t help at all.” He went on to say, “I can remember being mentally tired all the way back from when I was a kid” (P7). Several participants also expressed that they would like to exercise more, but that it was difficult when the dialysis treatment is 3 days per week and perhaps in addition to a job.

One participant said, “I need to find a way to deal with the exhaustion, that’s something I need to find a rhythm in before I can add exercise to my schedule” (P9).

### Hope and Hopelessness

Patients described their weight-loss endeavors with all-encompassing hopelessness, but the hopelessness was something they could rarely endure too long, and they would often turn it around to hope. Words expressing their feelings of having to lose weight included: impossible, battle, and that it simply was not going to happen. One participant said, “They told me to lose 10 kg, and my initial reaction was that I couldn’t, and that it wasn’t going to happen” (P1). Another participant found it basically impossible to lose weight: “When you have to lose more than 20 kg you might as well give up right away. It’s not realistic, and not something you just do in 6 months… I find it entirely impossible” (P6). Several participants described weight loss as something that required extra time and “room” for in life. They explained that they had too much going on currently to take on the weight-loss battle, and thought that when life settled down a bit, they would have the time to get serious about weight loss. One participant said, “When I first was told how much weight I needed to lose, it just knocked me completely out” yet went on to say, “I have actually already lost 12 kg, so I’m counting on that I can get on the list pretty soon” (P3). One participant was particularly frustrated with her inability to lose weight: “I’m so annoyed that I can’t just do it, I think to myself – why don’t you just do it?!” (P9).

### Support and Self-discipline

Support from the hospital, general practitioner, and the surrounding community along with close friends and family members was something the participants thought about a great deal, but they also viewed losing weight and generally taking care of themselves as entirely their responsibility, realizing that no one else could do the job for them. One participant thought the doctors and his family focused on his weight too much and felt like it took focus away from what was important, his kidney disease. He said that he had received a lot of support from the hospital where he had talked to a dietician but that he was not very good at following the advice he was given. Another participant said, “I see a dietician regularly, but honestly it’s your own responsibility to do something about it” (P5). One participant found weight-loss support on her own but also felt like she was on her own trying to figure it out. Another participant’s wife was very supportive when he wanted to go away on a 3-month weight-loss stay. He went on to explain that he had not received any support from the hospital or the local community. He said, “I was extremely frustrated when there was no help to get, but then I said, okay, only you can do something about this” (P1). One participant shared that the advice he got from a dietician was good, but that “it was basically the same old song; it’s easy to say what to do, but very hard to follow” (P6). Several participants were interested in having a group or a partner to meet with and exercise, someone to set goals with who would keep them motivated. One participant said, “I want someone to keep an eye on me, someone I meet with, so that if I show up and haven’t lost weight, that I would be embarrassed” (P4). Another participant said, “I would like to meet with someone and exercise. If I have to go to the gym alone, I just won’t get it done” (P6). Several participants found that the advice and diet plan they got from the dietician did not suit them very well and that it often suggested meals that they would never eat. One participant who was included in this study had already managed to lose sufficient weight to get on the transplant list; in response to a question asking him what it took, he only had one answer: “self-discipline and nothing else” (P1).

### Motivation Based on Severity

A general experience in this theme was that many of the participants did not feel much urgency to lose weight because their illness was not serious enough yet. However, they all declared that as soon as they got to be in very bad shape, they would have the motivation to lose sufficient weight. Other participants had discovered that losing weight was their only choice, simply because they were so hindered by their health in everyday life that they could not function at all. One participant stated that he was not motivated to lose weight yet because he did not feel ill enough. Another participant said that when she was told to lose weight, it completely flattened her, but she also said, “I’ve been thinking lately, that if I’m going to get a transplant at all, I’ll have to get on the weight loss very soon” (P3). One participant knew that she could keep her illness at bay, at least for a while, if she would eat healthily and exercise, but that dialysis was inevitable and transplant after that. Another participant related that he was so overweight that he could not do anything at all, and that is what eventually ended up motivating him to lose weight. He said, “It wasn’t until I started in dialysis that it became serious to me, when you suddenly can’t do anything at all, it’s time to get in gear” (P5).

## Discussion

In this semistructured interview study, we explored the thoughts, feelings, and attitudes of patients with obesity who must lose weight to receive a kidney transplant. Four themes arose from the interviews with the patients: (1) restrictions and exhaustion, (2) hope and hopelessness, (3) support and self-discipline, and (4) motivation based on severity.

Restrictions and exhaustion described how important food was to the participants in the study. Other studies have found food to be important as well.^21^, ^22^, ^23^ What to eat, when, and how much is extremely important when trying to lose weight. The restricted diet that is associated with both CKD and kidney failure was brought up by the participants repeatedly. A kidney-friendly diet, which consists of many modifications, is considered one of the most complex and restrictive therapeutic diets. In addition, adults with kidney failure tend to perceive the diet as complicated and contradictory to typical healthy eating advice. For example, fruits, vegetables, and dairy products are often restricted in kidney failure patients because of their potassium and/or phosphate content.^24^ Another element described in this theme was the level of exhaustion felt by the patients. Fatigue is often a major problem in patients with kidney disease and is seen in 60%-97% of patients with kidney failure undergoing hemodialysis.^25^ It has been reported to be a major obstacle to maintaining usual daily activities and quality of life.^26^ There is a strong association between the presence of fatigue and increased BMI, number of comorbid conditions, decreased physical activity, and depression. Furthermore, the severity of fatigue correlates with an increasing BMI.^27^ Extreme daytime sleepiness also correlates with an increased BMI, which is believed to be associated with both sleep apnea and a high-fat diet, which is shown to result in hypersomnolence in both humans and animals.^28^

Hope and hopelessness were found to be important experiences for the patients. Addressing pathways to sustained weight loss would often be met with a deep sense of hopelessness, but as the discussion continued it would be turned around to hope and a belief that things would improve, that weight loss would be obtained and thereby improve the patients’ physical condition. Hope is described as an optimistic attitude of mind based on an expectation of positive outcomes. Studies have found that hope is positively correlated with life satisfaction and serves as a buffer against the impact of negative and stressful life events.^29^ Studies have demonstrated that family members can help induce hope in severely ill patients and that this alone can help the affected patient to persevere.^30^ This can be applied to the participants in this study as well, both that they had a deep sense that things would get better, but also that they realized that their friends and family wanted them to get better and wanted them to lose sufficient weight. It is clear that hope is important and necessary to overcome difficulties in life.^29^

The theme support and self-discipline addressed how participants felt support or lack thereof in losing weight and developed enough self-discipline to stick with their weight-loss path. Lack of support, particularly from health care professionals, is described often in the literature. General practitioners often dismiss their patient’s obesity as a behavioral problem. It is assumed that weight loss is within a person’s control and that all they basically need to do is to eat healthily and exercise. It has been proposed that health care professionals are not interested in the patient’s personal story and that they do not recognize what the patient has already done and is currently doing.^22^^,^^31^^,^^32^ One study found that intense weight-loss counseling helps patients reach their goals.^15^ Self-discipline was described as an important ability and is often mentioned in the literature as the key to sustainable weight loss.^33^ It was evident from this study that some patients have the ability to be disciplined about their weight loss while others need more assistance. Individuals of healthy weight and those who can maintain their weight loss have been described as extremely vigilant about their weight, particularly regarding dietary intake. In contrast, weight regainers tend to feel that the effort involved in weight maintenance is not worthwhile.^21^

The theme motivation based on severity described the delaying of weight loss attempts. Reasons were numerous; too many things going on, too little time, too many hours at work, recently started dialysis, and having to move were some of the examples. Other literature has described the same; patients expressing contradictory feelings, and needing to get past the ambivalence to an acceptance that they have a responsibility to take action.^34^ Additionally, individuals that engage in little or no physical activity have a perceived lower quality of life. However, spending more time on physical activity will improve the quality of life and counteract procrastination.^35^ In more recent literature an increasing concern centers around socioemotional skills, especially self-control and self-regulation, with consequential poor health outcomes including obesity. Individuals with higher hyperbolic rates of discounting (hyperbolic discounting is a person’s desire for an immediate reward rather than a higher-valued, delayed reward) are considered impulsive or lacking self-control, tend to eat unhealthy food, drink too much alcohol, and procrastinate when it comes to engaging in healthy behaviors, which together led to obesity.^36^, ^37^, ^38^ The inability to obtain sufficient weight loss often denies patients with kidney failure the possibility of being placed on the kidney transplant list. If they do make it onto the transplant list, their weight may still keep them from receiving a kidney transplant.^12^ For this group of patients, weight loss must be achieved, and bariatric surgery before kidney transplant may be a possible way to ensure that eligible patients get their much-needed kidney transplant.^5^^,^^13^

The study has some limitations. Thematic saturation was reached after an unexpectedly low number of participants. We acknowledge that the inclusion of more participants could potentially have resulted in a deeper description of the individual themes. The patients were only interviewed once and over the phone, which may have lowered their confidence in describing the difficulties in something as personal as weight issues, although it has been shown that interviews over the phone can give participants a feeling of confidence to share their inner thoughts.^39^ Our interviews were relatively short (13 minutes on average), and this may have limited the depth of the conversation, although previous studies have shown that interviews over the phone tend to be shorter than in person.^40^

Patients from multiple hospitals should be included in future research to discover whether the findings of this study hold true for a wider group of participants. Future studies examining where the patients are on their weight-loss trajectory could prove beneficial to design weight-loss interventions that are better suited to the individual patient. In the future, improved dietary guidelines would benefit patients with kidney disease who need to lose weight.

In this study, we found 4 themes: (1) restrictions and exhaustion, (2) hope and hopelessness, (3) support and self-discipline, and (4) motivation based on severity. We explored the perspectives of the participants and discovered that they all desperately want to lose weight. They are painfully aware that losing weight is necessary to get a kidney transplant. The participants expressed that they want help to eat healthier and within the restrictions that come with their kidney disease. They want support from their surrounding community to exercise more, and they do not want to take on the weight-loss battle on their own. They want a partner or a group that can keep them motivated. Additionally, health care professionals should in general be educated to offer more and better support to their patients who are trying to lose weight, and physicians should be more proactive in offering obesity management medications and metabolic surgery options to these patients.
